# Governing Patient-Facing AI-Generated Video in Digital Health: A Risk-and-Ethics Matrix for Deployment, Monitoring, and Change Control

**DOI:** 10.2196/91940

**Published:** 2026-05-08

**Authors:** Yongzheng Hu, Wei Jiang

**Affiliations:** 1 Department of Nephrology, Affiliated Hospital of Qingdao University, 16 Jiangsu Road, Shinan District, Qingdao, Shandong, 266000, China, 86 13044087725; 2 Department of Nephrology, Qingdao University, Qingdao, Shandong, China

**Keywords:** artificial intelligence, AI, digital health, deepfakes, risk management, postdeployment monitoring

## Abstract

In this Viewpoint, we argue that patient-facing high-fidelity artificial intelligence (AI)–generated video requires governance that is operational, life cycle based, and embedded in existing institutional review pathways rather than limited to predeployment checks alone. Patient-facing high-fidelity AI-generated video—synthetic or substantially AI-mediated video that presents realistic human likeness, voices, or clinical communication cues—is rapidly entering patient education and clinical communication. We propose a risk-and-ethics matrix that combines residual clinical risk (likelihood × severity after mitigations) with an ethical alignment score that operationalizes autonomy, beneficence, nonmaleficence, and justice to yield actionable dispositions (encourage, permit with oversight, restrict or redesign, or prohibit). The framework links each disposition to dossier-based review, minimum controls, and postdeployment monitoring triggers—focused on measurable outcomes (eg, comprehension, content-attributable follow-up burden, incidents and complaints, and equity gaps) as well as provenance and change control—to support auditable, revisitable decisions over the system life cycle.

## Introduction

High-fidelity artificial intelligence (AI)–generated video—from text-to-video patient explainers to deepfake-style clinician avatars—is entering digital health via patient portals, telehealth workflows, and social platforms [1,2]. In this Viewpoint, we use this term to refer to synthetic or substantially AI-mediated video that presents realistic human likeness, voice, or other clinically salient communication cues in ways that may influence patient trust, comprehension, or decisions. We use “operational governance” to mean the institutional processes through which such systems are reviewed, approved, monitored, and re-evaluated over time. “Real-world deployment” refers to routine use outside controlled testing environments, including use through patient portals, telehealth workflows, apps, and social media channels where content may be redistributed or consumed without direct clinician mediation. “Iterative system change” refers to postdeployment modifications to models, prompts, templates, scripts, rendering pipelines, distribution channels, or disclosure and provenance controls that may materially alter system behavior. Early published examples suggest potential value for patient education and communication, including usability-tested patient digital twins for critical care education, avatar-based educational interventions associated with improved parental knowledge and care skills in hydrocephalus, and pilot or specialty use cases in radiology and postoperative patient communication [3-6]. However, governance often lags behind routine deployment, where content is redistributed across channels and iteratively updated (model, prompt, or template changes). These deployments are judged by outcomes beyond technical accuracy. Real-world clinical performance should be assessed using measurable end points that capture patient and system impact, including comprehension (eg, teach back checks), content-attributable follow-up burden, incident and complaint rates, and equity gaps across language and health literacy groups. This gap motivates a workflow-integrated approach that links upfront review to postdeployment monitoring, incident response, and change control across the life cycle [7,8].

The same properties that make AI-generated video attractive in digital health—realism, personalization, and rapid iteration—also create failure modes that are difficult to detect and manage once content is deployed across heterogeneous channels [9,10]. In routine settings, videos may be reposted or clipped outside institutional portals, updated as models and prompt templates change, and consumed without clinician context—conditions that allow for small errors to propagate into clinically consequential misinformation, unnecessary follow-up burden, or delayed care [11-13]. Identity cues embedded in video (eg, clinician likeness, institutional branding, or emotionally resonant avatars) can amplify perceived authority and trust, increasing the impact of misstatements, undisclosed synthetic identity, and privacy misuse. Before introducing residual clinical risk, it is important to distinguish it from clinical risk more broadly. In this paper, “clinical risk” refers to the possibility that patient-facing AI-generated video contributes to clinically relevant harm, including misinformation that changes care, delayed help seeking, unnecessary follow-up burden, privacy or identity misuse, psychological distress, or inequitable performance across patient groups. Risk becomes residual when these foreseeable failure modes are reassessed after proposed safeguards—such as clinician script review, constrained generation, authenticated distribution, disclosure, provenance controls, and escalation pathways—have been specified. Therefore, the matrix classifies the clinically relevant risk that remains after mitigation rather than the unmitigated theoretical hazard.

We propose a workflow-integrated governance approach: the risk-and-ethics matrix. It links residual clinical risk—defined here as the remaining likelihood and severity of clinically relevant harm after mitigation—to a principlism-based ethical alignment score (EAS) to support deployment decisions for patient-facing AI-generated video. Plotting these dimensions yields actionable dispositions (encourage, permit with oversight, restrict or redesign, and prohibit) and links each to minimum controls—dossier-based documentation, disclosure requirements, and human oversight where appropriate—as well as predefined postdeployment monitoring metrics and rereview triggers. We use representative scenarios to show how health systems can translate ethical commitments and probabilistic harms into auditable, revisitable decisions across the life cycle, particularly as content is updated and redistributed beyond its original workflow. We then summarize key risk mechanisms, present the scoring rubric and workflow, and map representative use cases of monitoring and change control actions.

The framework is intended for institutional decision-makers rather than for platform-wide moderation. In this paper, the relevant videos are those created, commissioned, adapted, or sponsored for patient-facing use. Typical producers include health systems; clinicians; patient education teams; digital health vendors; and researchers working in care delivery, education, or protocolized specialist settings. The primary governing bodies are local institutional actors such as institutional review boards (IRBs), digital health or clinical governance committees, patient education and communications leaders, and safety and IT oversight teams. Their role is to decide whether a proposed use case should be approved, under what minimum controls, and with what monitoring and rereview conditions. Existing laws, policies, and AI governance frameworks remain essential, but they often operate at a higher level of abstraction and do not specify how institutions should translate transparency, consent, safety, equity, provenance, and change control expectations into case-level deployment decisions for patient-facing AI-generated video use. What the matrix adds is not a replacement for law or formal regulation but an operational layer for institutional decision-making. It converts broad requirements such as transparency, human oversight, safety, equity, provenance, and accountability into case-level classifications, minimum controls, and life cycle management actions for specific patient-facing AI-generated video use cases [14].

The aim of this Viewpoint is to argue that governance of patient-facing AI-generated video should connect residual risk assessment and ethical alignment to concrete institutional decisions, life cycle monitoring, and change control. To support this argument, we outline the main risk mechanisms; present the risk-and-ethics matrix and its workflow; and then discuss implementation, validation, and adaptation across institutional and regulatory contexts.

## Risk Mechanisms in Routine Digital Health Deployment of AI-Generated Video

When AI-generated video is deployed routinely across patient portals, telehealth workflows, and social platforms, failure modes emerge that are not well captured by predeployment validation and, therefore, require postdeployment monitoring, incident response, and change control. First, misinformation and content or performance drift (eg, model updates, guideline changes, prompt template changes, and channel shifts) pose direct hazards to patient decision-making [15]. Hyperrealistic “clinician” avatars can convey inaccurate advice with a credibility premium that textual chatbots rarely command. Subtle script hallucinations and the lack of standardized clinical review workflows in many deployments amplify the chance that viewers will act on falsehoods before clinicians can intervene [16,17]. These risks are heightened in asynchronous, public-facing channels where corrections lag behind dissemination and platform ranking may preferentially surface engaging content.

Second, identity misuse and privacy infringements are uniquely salient when the video is the medium because identity cues drive trust calibration and downstream adherence. Unauthorized cloning of a clinician or patient’s likeness undermines autonomy, consent, and informational self-determination. Even ostensibly therapeutic recreations such as those involving deceased relatives raise unresolved questions about posthumous privacy and family interests [18,19]. Because mere visual plausibility confers trust, impersonation can catalyze fraud and degrade the informational environment far beyond the index case.

Third, psychological impact is bidirectional and context dependent. Video immersive qualities can deepen engagement, although they may also retraumatize, induce overattachment to synthetic figures, or blur boundaries between memory and simulation in grief and trauma work [20,21]. Minimizing these harms requires careful screening, clear framing, and predefined discontinuation criteria with escalation pathways to human care—not only technical guardrails.

Operationalizing these concerns should prioritize minimal, measurable guardrails. For comprehension and misinformation, institutions should track user-reported confusion, unplanned follow-up contacts attributable to the content, and brief comprehension checks (eg, teach back–style questions) in representative patient groups [22]. For identity and autonomy, all patient-facing deployments should meet a baseline disclosure standard, including clear labeling of synthetic content, explicit affirmation that no real clinician is speaking, and an accessible opt out. Psychological risk warrants prescreening, short validated distress scales, and predefined stop rules. Equity should be audited through stratified analyses of comprehension, incident or complaint rates, and follow-up burden (eg, by language, age, and health literacy). These metrics make benefits and harms visible enough to guide iterative redesign and trigger rereviews when thresholds are crossed.

Fourth, authenticity and institutional trust are collective goods at stake. As synthetic media saturate telehealth, patients may begin to doubt legitimate communications (“Is this my doctor or an AI?”) [23,24]. The resulting frictions—hesitation to follow instructions and demand for redundant confirmation—impose hidden costs on clinicians and organizations. Therefore, provenance signals and disclosure norms matter not as mere formalities but as trust-preserving infrastructure.

Finally, justice and equity considerations cut across all preceding risks. Benefits may accrue first to well-resourced settings that can build multilingual, culturally attuned avatars, whereas harms—deception, confusion, and exploitation—disproportionately fall on groups with lower health literacy or access to verification tools [25,26]. Thus, equity-oriented design, performance disaggregation, and complaint path accessibility are ethical requirements, not optional enhancements.

## Operational Governance Framework: The Risk-And-Ethics Matrix

We present an operational governance framework—the risk-and-ethics matrix—that supports deployment decisions for patient-facing AI-generated video by linking residual clinical risk to a principlism-based EAS and to predefined monitoring and rereview actions. Existing laws, policies, and AI governance frameworks establish essential high-level expectations. They do not usually specify how institutions should adjudicate a concrete patient-facing AI-generated video use case, what minimum controls should accompany approval, or when iterative changes should trigger rereview. Purely technical risk scoring tends to underweight autonomy and justice [27,28], whereas principle-first approaches can ignore how likely and severe harms are in actual practice [28-31]. Our integration preserves the strengths of both approaches and translates them into decisions that IRBs, hospital digital health and patient education governance groups, communications leaders, and safety and IT oversight committees can defend, document, and audit.

Here, we distinguish inherent or unmitigated clinical hazard from residual clinical risk, which is the basis for governance classification. On the risk axis, we adapt a hospital-grade matrix consistent with common clinical risk management concepts in which risk reflects the combination of probability and severity. For each use case, we score (1) the likelihood that a specified harm scenario will occur on a 4-level ordinal scale (rare, unlikely, possible, and likely) and (2) the severity of plausible consequences on a 4-level scale (negligible, minor, major, and catastrophic). Cross-tabulation yields composite tiers of low, moderate, high, and extreme residual risk. Assessment proceeds by enumerating the failure modes specific to synthetic video—misinformation that could change care, identity or privacy breaches, psychologically triggering content, or equity harms [19,32,33]—and then rating each mode and assigning the overall tier according to the highest credible residual risk after proposed mitigations. Mitigations—such as clinician review of scripts, constrained generation, authenticated distribution, and provenance or disclosure controls—are recorded in the dossier with versioning and change logs so that residual (not theoretical) risk is the basis of classification over time.

On the ethics side, we operationalize the 4 principles—autonomy, beneficence, nonmaleficence, and justice—into an EAS ranging from 0 to 8. Each principle receives a score of 0 when violated, a score of 1 when partially upheld, and a score of 2 when clearly upheld, guided by concrete criteria that map abstract duties to observable practices. Autonomy considers the transparency of AI use, the accuracy of identity representation, voluntariness, and the adequacy of consent in a video medium [34]; beneficence requires a credible, evidence-informed benefit that is proportionate to the foreseeable burdens [35]; nonmaleficence emphasizes minimizing physical, psychological, informational, and reputational harms and guarding against foreseeable misuse [36]; and justice attends to equitable access and performance across groups, bias mitigation, nonexploitation of vulnerable populations, and preservation of public trust [37]. We band the EAS scores as high (7-8), medium (4-6), or low (0-3); where evidence is limited, conservative scoring and explicit uncertainty statements are needed. Table 1 shows the scales and rubric.

**Table 1.** Scales and rubric for the risk-and-ethics matrix.

Component and level or principle	Definition or criterion
Likelihood	
Rare	Rare under routine conditions; requires multiple safeguards to fail
Unlikely	Single lapse or unusual context
Possible	Common precursor conditions present
Likely	Reproducible under routine conditions
Severity	
Negligible	No decision impact; self-correcting (eg, brief uncertainty without behavior change)
Minor	Transient confusion; extra contact (eg, 1 follow-up call or portal message for clarification)
Major	Clinically consequential misinformation or marked psychological harm (eg, delayed care, inappropriate self-management, or significant distress requiring clinician intervention)
Catastrophic	Severe harm or system-level misinformation (eg, serious injury, widespread harmful misinformation, or crisis-level psychological destabilization)
EAS^a^—autonomy	
0	No or unclear disclosure; misleading identity; no opt out
1	Disclosure present but incomplete or hard to understand
2	Clear disclosure; accurate identity; voluntary, informed consent
EAS—beneficence	
0	No credible benefit
1	Plausible benefit; limited evidence
2	Evidence-informed benefit; proportional to burdens
EAS—nonmaleficence	
0	Foreseeable significant harms
1	Harms possible with partial mitigation
2	Robust mitigation (human in the loop, constrained generation, or crisis plan)
EAS—justice	
0	Exacerbates inequity or exploitation
1	Neutral or unclear
2	Equitable access; bias mitigation; accessible complaint path
EAS banding	
High	7-8
Medium	4-6
Low	0-3

a EAS: ethical alignment score.

Residual clinical risk is scored on a 4-level likelihood scale (rare, unlikely, possible, and likely) and a 4-level severity scale (negligible, minor, major, and catastrophic) interpreted after proposed mitigations. Ethical alignment is scored using the EAS (0-8), operationalizing autonomy, beneficence, nonmaleficence, and justice on a scale from 0 to 2 per principle and then banded as high (7-8), medium (4-6), or low (0-3). Ratings should reflect residual (not theoretical) risk, with conservative defaults and explicitly recorded uncertainty when evidence is sparse, and should be versioned for rereview after material changes.

**Figure 1.**
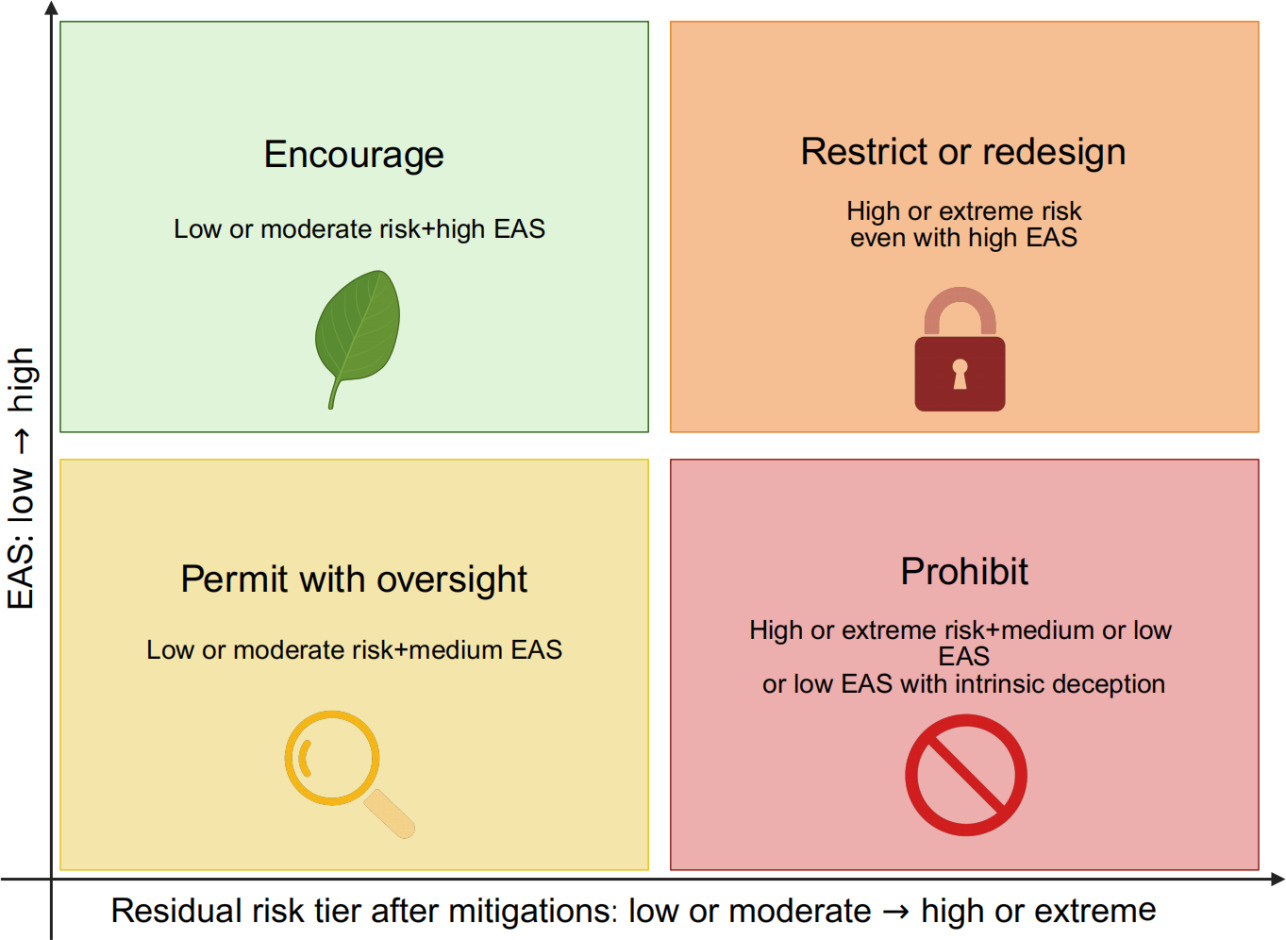
Risk-and-ethics matrix for patient-facing generative artificial intelligence video in health care. Residual clinical risk (likelihood × severity after mitigations) is plotted against ethical alignment (ethical alignment score; EAS), yielding 4 deployment dispositions: encourage, permit with oversight, restrict or redesign, and prohibit. The horizontal axis represents the residual risk tier (low, moderate, high, or extreme), and the vertical axis represents EAS band (high, medium, and low), where EAS operationalizes autonomy, beneficence, nonmaleficence, and justice on a scale from 0 to 8. Entry rules prioritize residual (not theoretical) risk and link each disposition to minimum controls and rereview triggers.

Plotting the residual risk tier against EAS bands yields 4 deployment dispositions with explicit entry rules (Figure 1): encourage, permit with oversight, restrict or redesign, and prohibit. “Encourage” applies when residual risk is low and ethical alignment is high, supporting routine deployment with disclosure and periodic quality assurance. “Permit with oversight” covers moderate risk with at least medium ethical alignment (or low risk with medium ethical alignment) and requires human-in-the-loop review where appropriate, audit trails, incident reporting, time-limited approvals, and a postdeployment monitoring plan with predefined triggers for rereview. “Restrict or redesign” is appropriate when residual risk is high or when ethical alignment is low in the absence of intrinsic deception (ie, the use case is not fundamentally based on impersonation, undisclosed synthetic clinicians, or manipulative identity cues); here, scope narrowing, stronger transparency and safety guardrails, and protocolized pilots are prerequisites for reconsideration. “Prohibit” is reserved for extreme risk or for low ethical alignment tied to intrinsic deception or manipulation that exploits vulnerabilities; in such cases, deployment is disallowed and takedown, reporting, and other platform- or legal-level remedies may be warranted. These thresholds aim less for numerical precision than for defensible consistency across cases and clarity about what concrete changes would move an application toward a safer, more ethically aligned quadrant.

To support consistency, the rubric anchors abstract principles to concrete artifacts (eg, disclosure language, escalation pathways, evidence of benefit, and provenance controls) so that different panels can converge even when data are sparse [38,39]. Interrater reliability is promoted through independent prescoring, structured reconciliation, and written rationales for deviations from precedent. Because both risk and ethics are provisional in fast-moving sociotechnical contexts [40], institutions should version scores with date-stamped assumptions and require rereview after model updates, distribution channel changes, or sentinel events. Therefore, classification is not a verdict but a living record of judgment under stated conditions.

**Figure 2.**
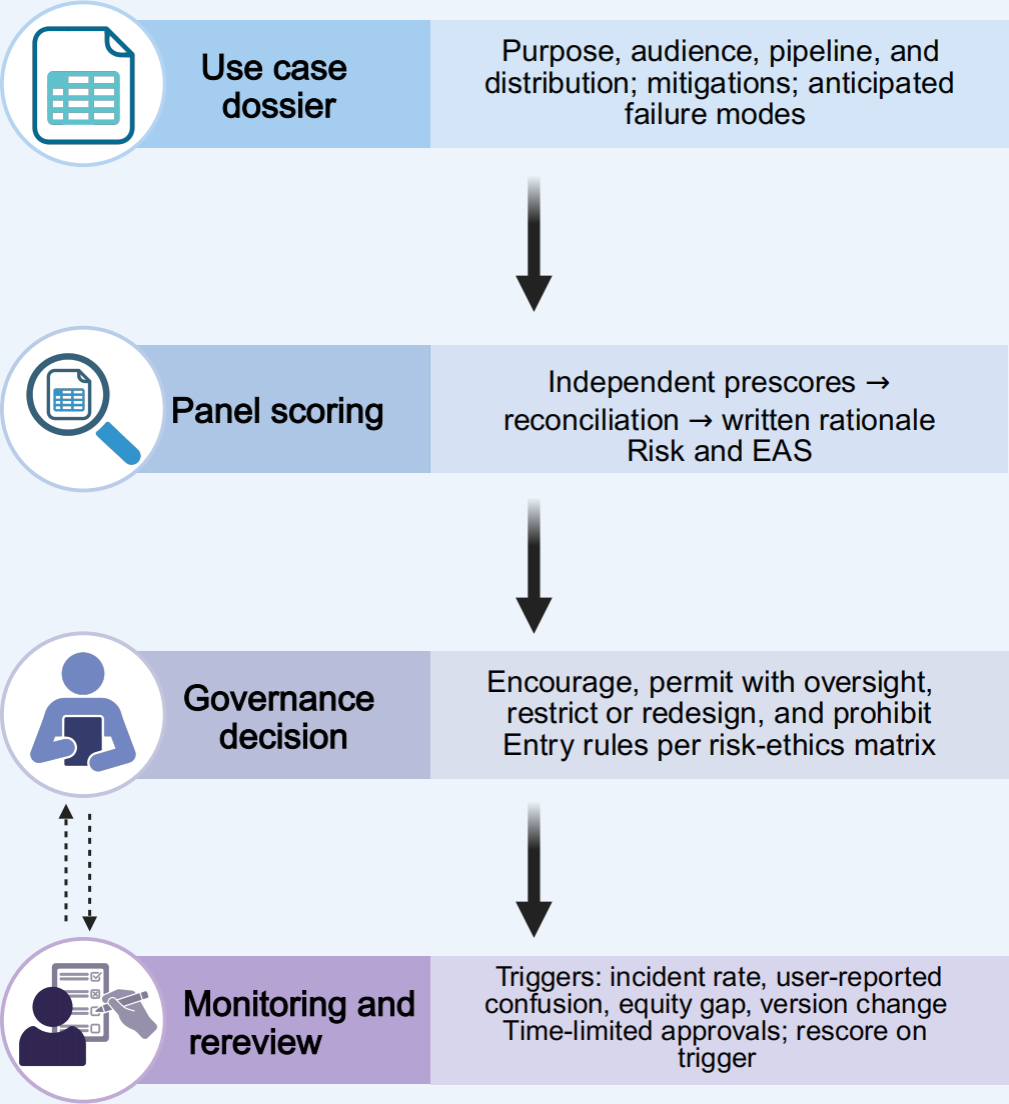
Workflow from use case dossier to classification and postdeployment monitoring. Proponents submit a versioned use case dossier; a triad panel (clinician or health educator, bioethicist, and safety or IT lead) independently prescores residual risk and ethical alignment score (EAS), reconciles differences, and records a written rationale. The resulting disposition maps to minimum controls (Table 2) and a monitoring plan with predefined indicators and rereview triggers (eg, incidents, user-reported confusion, equity gaps, and version changes). Operational checklist and monitoring triggers are provided in Multimedia Appendix 1; the dossier template is provided in Multimedia Appendix 2.

Finally, we specify a lightweight workflow that fits existing governance rather than creating parallel structures. Because review burden should be proportional to risk and novelty, we do not assume a single fixed evaluation time for all use cases. Low-risk, template-based, clinician-vetted educational videos that closely follow prior approved formats may undergo an expedited review focused on dossier updates, disclosure, and any material changes, whereas novel, higher-risk, psychologically sensitive, identity-based, or publicly disseminated use cases warrant fuller panel deliberation and documentation. Proponents submit a use case dossier describing purpose and audience, generation pipeline, distribution channel, mitigation plan, anticipated failure modes, and versioning or change logs. A triad panel—a clinician or health educator, bioethicist, and safety or IT lead—scores risk and EAS independently, reconciles differences, and documents residual disagreements; panels may co-opt patient advocacy or health literacy expertise for patient-facing deployments when needed. Decisions link directly to the chosen disposition and to a postdeployment monitoring plan with predefined indicators (eg, misinformation incidents, user-reported confusion, complaint rates, follow-up burden, and equity gaps) and time-bound rereview triggers. Operational steps are summarized in Figure 2; a printable evaluator’s checklist and monitoring triggers can be found in Multimedia Appendix 1, and a structured use case dossier template can be found in Multimedia Appendix 2. For institutional use, matrix-guided evaluation should be embedded into release governance such that publication or distribution through official channels requires completed dossier documentation, named sign-off, and versioned approval records. Materials that bypass review or breach minimum controls should trigger withholding of institutional dissemination; pause or takedown; incident review; and, where applicable, corrective action under local policy. In this sense, the key incentive structure is not speed-based reward. It is the linkage of authorization, traceability, and consequences to the right to deploy patient-facing AI-generated video. Where feasible, dossiers should trace content provenance from prompt to rendered asset to distribution and specify abuse-resistant defaults (eg, prohibited impersonation classes and persistent labeling), with escalation procedures when out-of-distribution use or unequal performance emerges. Table 2 shows governance categories and minimum controls.

**Table 2.** Deployment dispositions, entry rules, and minimum controls.

Category	Residual risk tier × EAS^a^ band	Minimum controls^b^	Illustrative examples^c^
Encourage	Low risk and high EAS	Disclosure and routine QA^d^	Transparent, clinician-vetted patient education avatar
Permit with oversight	Moderate risk and ≥medium EAS or low risk and medium EAS	Human in the loop, audit trail, incident reporting, and time-limited approval	Protocolized “deepfake therapy” in specialist or IRB^e^ settings
Restrict or redesign	High risk or low EAS (no intrinsic deception)	Scope narrowing, stronger transparency and safety, and piloting then rescoring	Free-text explainer without clear disclosure
Prohibit	Extreme risk or low EAS with intrinsic deception or manipulation	Takedown and platform- or legal-level remedies	Impersonated physician endorsements

a EAS: ethical alignment score.

b Minimum controls specify documentation, human oversight, incident reporting, time-limited approval, and rereview triggers as applicable.

c Examples illustrate typical placements.

d QA: quality assurance.

e IRB: institutional review board.

## Use Case Mapping: From Dossier Evidence to Monitoring Plans

The following use cases illustrate a repeatable mapping from dossier evidence to residual risk or EAS scoring, a deployment disposition, and a minimal monitoring plan with explicit triggers for rereview.

### Case 1: Patient Education Avatar (Transparent, Vetted, and Multilingual)

A hospital deploys short, tailored videos explaining procedures and postoperative care through a clearly disclosed AI avatar delivered via an authenticated patient portal. Scripts are clinician vetted, linguistically and culturally adapted, and version controlled. The portal supports replay, adjustable playback speed, and an easy pathway to request human follow-up or report confusion. Residual risk is low: under constrained scripts plus clinical review and secure distribution, misinformation is rare or unlikely and typically minor (eg, transient confusion or extra contact), and privacy exposure is limited because the content is generic. Ethical alignment would likely be high (autonomy=2, beneficence=2, nonmaleficence=2, and justice=2): disclosure and an opt out support autonomy, measurable gains in comprehension and reduced avoidable follow-up burden support beneficence, constrained generation and review reduce foreseeable harms, and multilingual access advances justice. The resulting disposition is to encourage, with routine quality assurance and a time-bounded review cadence plus rereview triggers for guideline changes, prompt template updates, channel changes, or stratified performance gaps across language and health literacy groups [41,42].

### Case 2: “Deepfake Therapy” for Grief (Therapist Led, Protocolized, and Time Limited)

Under IRB-approved protocols, a psychotherapist offers a time-bounded intervention in which a synthetic likeness of a deceased relative delivers scripted messages to facilitate goodbye rituals. A research or specialist setting matters because it enables structured screening, standardized outcome capture, adverse event reporting, and enforceable stop rules. Residual risk is moderate to high: even with careful preparation, clinically meaningful psychological harms may occur (severity level: major), and the likelihood may be possible or likely in vulnerable subgroups. Ethical alignment would likely be medium (autonomy=1, beneficence=2, nonmaleficence=1, and justice=1): beneficence may be credible for selected patients; autonomy depends on robust consent that frames the video as a simulation and checks understanding; nonmaleficence relies on screening, therapist presence, predefined discontinuation criteria, and escalation pathways; and justice requires equitable access criteria and avoidance of coercive commercialization. The resulting disposition is to permit with oversight only in research or specialist settings, with predefined outcome measures (including follow-up windows), incident logging, and immediate cessation upon adverse reactions; approvals should be time limited, with rereview triggers tied to protocol deviations, adverse events, or material changes to the model or pipeline [5,43,44].

### Case 3: Impersonated Physician Endorsements (No Consent and Public Dissemination)

A synthetic clone of a prominent clinician appears on social media platforms to promote unverified health products or claims. Residual risk is extreme: harm is likely because the video exploits identity-based trust and can divert patients from evidence-based care; severity level is major to catastrophic if it prompts medication changes, delays appropriate care, or amplifies misinformation at scale. Ethical alignment would score low across all 4 principles: deception negates autonomy; benefits are not patient centered; harms are foreseeable and unmitigated; and the practice exploits vulnerable audiences, undermining justice and public trust. The resulting disposition is to prohibit. Rapid takedown and reporting workflows, provenance checks, public clarification through verified institutional channels, and legal remedies are warranted; organizations should precommit to a zero-tolerance policy for unauthorized likeness use and log incidents to strengthen postmarket monitoring and prevention [44-46].

## Discussion

### Implications for Digital Health Implementation

Our risk-and-ethics matrix translates life cycle AI governance expectations into a practical format for clinical decision-makers by pairing probabilistic risk appraisal with principled ethics in a way that is explicit, documentable, and revisitable. The distinctive governance value of the risk-and-ethics matrix is that it bridges the gap between high-level regulatory expectations and local operational decisions. Rather than offering another abstract set of principles, it enables institutions to classify a specific use case; document the rationale for approval or restriction; assign minimum controls; and connect deployment to monitoring, incident response, and change control over time. This orientation is consistent with major governance frameworks that emphasize postdeployment monitoring, mechanisms for capturing user input, incident response, and change management as core components of responsible AI use in real-world settings [47]. We localize these expectations to patient-facing generative AI video by grounding “risk tiering” in concrete clinical harm scenarios (eg, misinformation that changes care, identity misuse, psychological triggering content, and equity harms) and by converting principlism—autonomy, beneficence, nonmaleficence, and justice—into action-guiding criteria through an EAS. Together, the 2 axes yield implementable dispositions—encourage, permit with oversight, restrict or redesign, and prohibit—whose entry rules can be recorded, audited, and defended as part of evaluating real-world clinical performance.

Beyond classification, the matrix provides a shared structure for interdisciplinary deliberation and practical redesign. The framework also functions as a design instrument: because dossiers trace why a proposal lands in a given disposition, developers are directed toward concrete modifications—clear disclosure and comprehension-checked consent to strengthen autonomy; scope limitation, constrained generation, and human-in-the-loop review to reduce residual risk; and stratified monitoring to strengthen justice. In parallel, professional guidance on generative AI in medicine underscores the importance of preserving human oversight and aligning deployments with clinical workflows rather than displacing them—an emphasis that is especially salient for persuasive patient-facing media. Conversely, the matrix clarifies “red-line” cases grounded in intrinsic deception (eg, impersonated clinician endorsements), supporting rapid takedown, institutional clarification through verified channels, and incident logging to strengthen future prevention.

National-level safeguards become especially important in cases in which institutional incentives favor speed, visibility, or monetization over careful review. In China, the relevant governance architecture is emerging but remains distributed across health sector, platform, and generative AI rules. Existing measures already provide building blocks, including synthetic content labeling and traceability, filing and disclosure for certain public-facing AI services, and health sector expectations for account registration and monitoring. The next step is to connect these elements through sector-specific requirements for disclosure, provenance, verified identity, monitoring, rapid correction or takedown, and enforceable accountability. Such national-level guardrails would not replace local review tools such as the risk-and-ethics matrix; rather, they would create the incentive environment in which institutional review is more likely to be performed seriously and consistently.

Feasibility in smaller or resource-limited settings will depend on tiered implementation rather than assuming the full model from the outset. The core minimum is not a large committee but a documented review pathway with clearly assigned accountability, use case documentation, explicit disclosure checks, and a mechanism for escalation when risk exceeds local expertise. For familiar low-risk educational videos, institutions with limited resources may use a simplified pathway involving a clinically accountable reviewer plus a second reviewer with operational or technical oversight supported by a standardized checklist and basic postdeployment signals such as complaints, follow-up contacts, and disclosure failures. The fuller triad panel model—with dedicated bioethics input, richer analytics, formal incident reporting, and equity stratification—should be viewed as an expanded configuration for higher-risk or more mature settings. Where dedicated bioethics expertise is unavailable, regional ethics consortia, shared review pools, tele-ethics consultation, or referral pathways to larger centers may provide a practical alternative, especially for first-in-class, psychologically sensitive, identity-based, or publicly disseminated use cases.

### Limitations and Validation Agenda

This approach has limitations. Early deployments will often rely on expert judgment because empirical evidence on the frequency and magnitude of novel harms remains sparse, and both residual risk estimates and EAS components can vary with local context [48-51]. To temper subjectivity, we emphasize independent prescoring, structured reconciliation, and written rationales anchored to observable artifacts (eg, disclosure language, evidence of benefit, escalation pathways, and provenance controls) [52]. Because the EAS is intended as an operational rubric rather than a purely intuitive checklist, institutions should prospectively calibrate and evaluate its reliability before routine use. A practical approach would be to begin with a set of anchor case vignettes spanning low-, medium-, and high-alignment scenarios; require independent prescoring by panel members; conduct structured reconciliation with written reasons for disagreement; and repeat calibration periodically using shared cases across panels or sites [53]. For the ratings of 0 to 2 assigned to each principle, agreement could be summarized using percentage agreement and weighted κ, whereas the reliability of the summed EAS from 0 to 8 could be examined using an intraclass correlation coefficient. Institutions could additionally track agreement on EAS banding and on the final deployment disposition because these outputs are directly tied to governance decisions. Content validity could be strengthened through multidisciplinary expert review of whether the rubric adequately captures observable manifestations of autonomy, beneficence, nonmaleficence, and justice, with iterative refinement through pilot-testing or Delphi-style consensus procedures [54]. Construct validity could then be explored by testing whether the EAS discriminates between use cases that are expected a priori to differ in ethical alignment (eg, transparent clinician-vetted education avatars vs impersonated clinician endorsements) [55]. Importantly, classification should be treated as a living record—versioned with date-stamped assumptions—so that uncertainty becomes auditable and revisable rather than implicit [56,57]. The same use case may plausibly yield different profiles across clinical domains (eg, perioperative education vs mental health) or across resource settings; documenting contextual assumptions and applying prespecified domain modifiers can improve consistency without suppressing legitimate local variation. Finally, risk severity overlaps conceptually with nonmaleficence, and beneficence often embeds risk-benefit trade-offs [58,59]. Therefore, treating the axes as orthogonal is a usability heuristic—intended to promote clarity and reproducibility—rather than a claim of theoretical independence; cross-referencing during deliberation should be expected.

### Measurement, Monitoring, and Rereview Triggers

These caveats point to a concrete real-world evaluation agenda. Retrospective incident reviews and prospective pilots can stress test thresholds and calibrate rubrics using patient-centered and workflow-relevant end points (eg, comprehension or teach back performance, unplanned follow-up contacts attributable to content, complaint and incident rates, and stratified equity gaps). Textbox 1 summarizes a core monitoring set, operational data sources, and example rereview triggers that can be embedded into routine digital health workflows. Governance-relevant measurement should be paired with life cycle mechanisms for capturing user input and adjudicating overrides and with explicit incident response and recovery pathways—elements foregrounded in the National Institute of Standards and Technology’s risk management guidance for deployed AI systems [14]. Harmonization with provenance and disclosure standards can further improve auditability and reduce identity-related misuse, enabling versioned reassessments as models, prompts, distribution channels, and guardrails evolve. For systems that will undergo iterative change, “change control” should be planned rather than improvised; regulatory thinking around predetermined change control plans provides a useful template for specifying anticipated modifications and the evidence required to validate them over time. Comparative ethical analysis may also be valuable in edge cases where principlism and alternative lenses diverge; documenting such divergences can refine decision rules while preserving usability [60,61].

Textbox 1. **Core monitoring metrics, operational data sources, and rereview triggers for patient-facing artificial intelligence–generated video.**

**Core metrics (minimum set)**

- Comprehension: brief teach back–style checks or short postview questions and user-reported confusion
- Content-attributable follow-up burden: messages, calls, or telehealth follow-ups attributable to the video (eg, tagged reason codes or postview “contact clinician” clicks)
- Incidents and complaints: safety reports and formal complaints linked to the content (misinformation, identity misuse, or privacy concerns)
- Equity gaps: stratified differences in comprehension, follow-up burden, and incidents or complaints (eg, by language and health literacy proxies)
- Provenance and trust: visibility of synthetic content disclosure, verification friction (eg, “Is this my doctor?” queries), and confirmed impersonation attempts

**Data sources (digital health operations)**

- Patient portal and telehealth analytics (views, completion, and click-through to contact or obtain support)
- Secure messaging and call center logs (tags and reason codes and clinician note templates for attribution)
- Incident reporting and complaint systems (patient safety and privacy or security tickets)
- Patient feedback channels (postview survey and “report confusion/request human help” buttons)
- If distributed externally, verified channel monitoring (eg, takedown requests and platform reports)

**Rereview triggers (examples; adapt locally)**

- Material change: model, prompt or template, script or guideline, channel, or language rollout updates
- Signal excursion: sustained rise in follow-up burden or confusion reports vs baseline
- Safety event: any major incident or clustered minor incidents attributable to the content
- Equity flag: new or widening stratified gaps in end points
- Provenance or identity event: confirmed impersonation, unauthorized likeness use, or disclosure failure

In early deployments, trigger thresholds should be treated as provisional rather than fixed regulatory cutoff points. A pragmatic starting approach is to establish a local baseline during an initial pilot or first complete rollout cycle and then update thresholds iteratively as more observations accumulate. During this early phase, institutions may use structured expert consensus or Delphi-style calibration among early adopters to define provisional trigger ranges, with wider tolerance bands and explicit uncertainty notes until local rates stabilize. Thresholds should ideally be interpreted against rolling local baselines rather than in isolation and should be re-estimated after material workflow, model, channel, or language changes. For equity analyses in particular, subgroup differences should not be overinterpreted when denominators are sparse; institutions should prespecify minimum subgroup counts before treating observed gaps as decision relevant and, where counts remain small, use descriptive flagging and continued data collection rather than strong statistical conclusions.

Here, “rereview” denotes the procedural reassessment triggered by monitoring signals, incidents, or material changes. “Reclassification” denotes a substantive change in risk tier, EAS band, or deployment disposition that may result from that reassessment. Because ethical priorities and risk tolerance vary across cultures and health systems, the framework is designed to be portable yet tunable [62,63]. To preserve comparability while allowing for local adaptation, the framework distinguishes core elements that should remain stable across sites from parameters that may be tuned to local context (Table 3).

**Table 3.** Core standardized elements and locally tunable parameters of the risk-and-ethics matrix.

Domain	Core elements (preserve across sites)^a^	Tunable parameters (adapt locally)^b^
Ethical architecture	Four principles; 0-2 EAS^c^ scoring logic; high, medium, and low EAS banding	Local case anchors, examples, and training materials
Residual risk assessment	Residual risk logic after mitigation; 4-level likelihood and severity structure	Default risk modifiers for high-vulnerability domains or populations
Deployment decisions	Four disposition categories (encourage, permit with oversight, restrict or redesign, and prohibit)	Local approving body, escalation route, and implementation authority
Documentation and oversight	Use case dossier, rationale, versioning, accountability, and human oversight	Dossier format; full triad panel vs simplified or shared review pathway
Disclosure and provenance	Minimum synthetic content disclosure, identity accuracy, and provenance expectations	Disclosure wording, language level, format, and content credential implementation
Monitoring and rereview	Monitoring, incident capture, equity review, and rereview trigger logic	Indicator thresholds, baseline methods, observation windows, subgroup definitions, and language coverage minimums

a Core elements are intended to preserve conceptual and operational comparability across sites.

b Tunable parameters may be adapted to local workflow capacity, legal context, language needs, and risk tolerance provided that deviations are specified prospectively and documented consistently within the adopting institution.

c EAS: ethical alignment score.

Crucially, classification must remain revisitable. Approvals in “permit with oversight” should be time limited and coupled with predefined rereview triggers (eg, model or prompt updates, distribution channel changes, sentinel incidents, or emerging inequities). Successful mitigation and accumulating evidence may move a use case toward “encourage,” whereas incidents or drift may push it toward “restrict or redesign” or “prohibit.” Embedding this cadence operationalizes continuous risk management and aligns oversight with the broader shift toward postdeployment monitoring systems for AI in real-world settings.

The risk-and-ethics matrix is intended to complement, not replace, formal regulatory review. Its role is to translate broad regulatory expectations into case-level institutional governance for patient-facing AI-generated video use. In cross-jurisdictional terms, the framework’s disclosure, identity, and provenance elements align with transparency-oriented requirements; its human-in-the-loop review, escalation pathways, and authority to pause or withdraw deployments align with expectations around human oversight; its dossier, versioning, and documented rationale align with technical documentation and record-keeping expectations; and its monitoring indicators, incident triggers, and rereview cadence align with postmarket monitoring and iterative change control requirements. This means that, where a use case is already subject to sector-specific regulation—such as the European Union AI Act’s risk-based obligations for certain AI systems or medical device review pathways that incorporate predetermined change control planning—the matrix is not a substitute for those legal processes. Rather, it provides a local operational layer that helps institutions implement, document, and monitor responsible use under real-world conditions.

### Conclusions and Next Steps for Implementation

AI-generated video is becoming a routine modality for patient-facing communication, making life cycle governance essential to safe real-world deployment [1,64,65]. This Viewpoint presents an operational risk-and-ethics matrix that links residual clinical risk and ethical alignment to auditable, revisitable deployment decisions.

Future work should prioritize 3 deliverables. First, real-world evaluation should calibrate thresholds with patient-centered and workflow-relevant end points (eg, comprehension and teach back, content-attributable follow-up burden, incidents and complaints, and stratified equity gaps) and embed monitoring, incident response, and change management with time-limited approvals and rereview triggers. Second, provenance should be strengthened through clear disclosure and interoperable content credentials to reduce identity misuse and support verification. Third, change control should be planned rather than improvised: institutions should prespecify anticipated model, prompt, or pipeline updates and the evidence required to maintain assurance over time, drawing on the Food and Drug Administration’s predetermined change control plan approach for AI-enabled systems.

## Supplementary material

10.2196/91940 Multimedia Appendix 1 Operational tool pack (evaluator’s checklist and monitoring triggers). This pack operationalizes the risk-ethics matrix for routine governance. Part A provides a 10- to 12-item evaluator’s checklist. Part B lists monitoring indicators with example triggers. Crossing a trigger prompts rereview and reclassification per Table 2 and Figure 2.

10.2196/91940 Multimedia Appendix 2 Use case dossier template (versioned submission form). The dossier captures purpose and audience, generation pipeline, distribution channel, content and privacy, failure modes with mitigations, ethical alignment score (EAS) evidence (0-2 per principle), residual risk tier, governance ask, monitoring plan and thresholds, sign-offs, and change log. It aligns with Table 1 (scales and EAS rubric), Figure 1 (quadrant mapping), Table 2 (entry rules and minimum controls), and Figure 2 (workflow). Panels perform independent prescores, reconciliation, and written rationales; approvals in yellow zones are time limited, with predefined triggers for rereview.

## Abbreviations

AI: artificial intelligence
EAS: ethical alignment score
IRB: institutional review board
