# Supplementary material for: Governing Patient-Facing AI-Generated Video in Digital Health: A Risk-and-Ethics Matrix for Deployment, Monitoring, and Change Control
Source: J Med Internet Res. 2026 May 8;28:e91940. doi: 10.2196/91940 (PMC13155343; doi:10.2196/91940)
Supplement: Multimedia Appendix 1 [file jmir-v28-e91940-s001.doc]

# Operational Tools Pack

This appendix provides operational checklists and monitoring triggers to support implementation of the Risk–Ethics Matrix in routine digital health governance.

## Part A — Evaluator’s Checklist

Use this binary checklist before deploying any patient-facing generative video. Mark each item as **Yes/No**; resolve all “No” items (with owners and due dates) prior to approval.

☐ **Require** clear synthetic-content disclosure, including an on-screen label (and accompanying text where the video is posted)
☐ **Verify** identity representation is accurate; prohibit impersonation or likeness use without explicit consent and authorization
☐ **Document** human-in-the-loop (HITL) review for scripts and rendered assets (reviewer, date, version)
☐ **Enable** provenance/watermarking and metadata capture in the distribution pipeline (eg, Coalition for Content Provenance and Authenticity [C2PA] where feasible)
☐ **Document** data sources, rights/licensing, and privacy pathways (collection, storage, retention, access controls)
☐ **Pre-specify** an equity assessment plan (eg, language/age/health-literacy stratification)
☐ **Complete** a predeployment pilot (or rehearsal) and archive logs and outputs for audit
☐ **Configure** a monitoring dashboard with named indicators, baselines, and locally defined thresholds
☐ **Register** sentinel triggers in the governance system (what triggers re-review; who is notified; expected response time)
☐ **Record** time-limited approval with a re-review date (and conditions for early re-review)
☐ **Document** user support and escalation pathways (how users reach a human; how issues are triaged)
☐ **Enable** abuse-resistant defaults (eg, constrained generation; disallow impersonation classes; rate limits where applicable)

**Note:** Attach this checklist to the Use-Case Dossier at submission; track unresolved items with owners and timelines.

## Part B — Monitoring Indicators & Trigger Thresholds

- **Local calibration:** Thresholds (X, Y) should be set locally by domain (eg, perioperative education vs mental health), informed by baseline rates and risk tier; example triggers below are illustrative only.
- **Major incident definition:** Define “major incident” a priori (eg, clinically consequential misinformation, identity/privacy breach, or marked psychological harm) and log each event with root-cause categorization and corrective action.

| **Indicator** | **Definition** | **Data source** | **Trigger (example)** |
| --- | --- | --- | --- |
| Misinformation incident rate | Number of misinformation events per 1,000 video views (include confirmed vs suspected). | Patient safety reports; moderation flags; clinician reports; complaint logs. | Trigger: ≥1 major incident **or** sustained increase above baseline for a rolling 30-day window. |
| User-reported confusion | Proportion of viewers indicating confusion or requesting clarification postview. | Postview survey item; “report confusion” button; portal feedback forms. | Trigger: ≥X% reporting confusion in a rolling 30-day window. |
| Content-attributable follow-up burden | Follow-up contacts attributable to the content per 100 views (messages/calls/telehealth follow-ups). | Portal “contact clinician” click-through; secure messaging tags; call center reason codes; clinician note template flags. | Trigger: Spike beyond historical control limits **or** sustained rise above baseline for a rolling 30-day window. |
| Language-stratified comprehension | Comprehension score (eg, teach-back/quiz accuracy) by subgroup. | Embedded micro-quiz/teach-back check; structured follow-up documentation. | Trigger: Equity gap ≥Y percentage points (persistent across ≥2 measurement cycles). |
| Complaint ratio | Formal complaints per 1,000 views attributable to the content (misinformation, disclosure failure, privacy/identity concerns). | Patient relations logs; grievances; privacy/security ticketing. | Trigger: ≥X% within rolling 30 days **or** any cluster suggesting common failure mode. |
| Material change (automatic re-review) | Any material change to model, prompt/template, script/guideline basis, distribution channel, or disclosure/provenance controls. | Change-control log; release notes; governance ticketing system. | Trigger: Automatic re-review upon recorded change (time-limited approval may also mandate reclassification). |

Track these indicators postdeployment. Crossing any trigger should prompt re-review and, where warranted, a revised classification.

**Abbreviations:** HITL, human-in-the-loop; C2PA, Coalition for Content Provenance and Authenticity.
