# Supplementary material for: Governing Patient-Facing AI-Generated Video in Digital Health: A Risk-and-Ethics Matrix for Deployment, Monitoring, and Change Control
Source: J Med Internet Res. 2026 May 8;28:e91940. doi: 10.2196/91940 (PMC13155343; doi:10.2196/91940)
Supplement: Multimedia Appendix 2 [file jmir-v28-e91940-s002.doc]

# Use-Case Dossier Template

### Instructions

## **Provide** concise, evidence-backed responses. **Attach** the Evaluator’s Checklist (Multimedia Appendix 1) and relevant artifacts (scripts, screenshots, consent/disclosure language, sample outputs).

## **Assign** a unique dossier ID and **version** this dossier (version, date, owner).

## **Update and re-submit** upon any **material change** to the model, prompt/template, pipeline, distribution channel, or disclosure/provenance controls (see Change log).

## 1) Purpose & Audience

**Define** the clinical intent and intended users.

**Specify:**

1. Intended use (what the video is for) and intended audience (patient group; caregivers).
2. Use context (education vs behavior change vs decision support; routine vs high-stakes).
3. Contraindications/exclusion criteria (if any) and “do-not-use” contexts.

_______________________________________________

_______________________________________________

_______________________________________________

_______________________________________________

_______________________________________________

_______________________________________________

## 2) Generation pipeline

**Describe** how content is produced and reviewed.

**Specify:**

1. Model(s)/tools and versions; prompt/template identifiers; script source(s).
2. Human-in-the-loop (HITL) points (who reviews script, render, and final cut).
3. Roles and sign-offs (name/role; date; version reviewed).
4. Change-control plan: anticipated updates (model/prompt/template) and minimum validation evidence required before redeployment.

_______________________________________________

_______________________________________________

_______________________________________________

_______________________________________________

_______________________________________________

_______________________________________________

## 3) Distribution channel

**Define** where and how the video is delivered and controlled.

**Specify:**

1. Channel(s): portal/app/telehealth/social; authenticated vs public access.
2. Verified-channel measures (eg, account verification; access controls; link integrity).
3. Takedown/recall mechanism (owner; SLA; steps; how users are notified).
4. Content lifetime (expiration/review date) and archival policy.
5. Provenance/Content Credentials (eg, Coalition for Content Provenance and Authenticity [C2PA]): whether embedded; whether preserved downstream; how users can verify.

_______________________________________________

_______________________________________________

_______________________________________________

_______________________________________________

_______________________________________________

_______________________________________________

## 4) Content & Privacy

**Document** content sources, permissions, and data handling.

**Specify:**

1. Source media and rights/licensing (including any clinician likeness/voice).
2. Consent approach (who consents; when; how recorded) and exact disclosure text (copy-paste).
3. Personally identifiable information (PII) handling: data minimization; storage location; access control; retention/deletion timeline.
4. Incident reporting contact (privacy/security; patient safety) and reporting route.

_______________________________________________

_______________________________________________

_______________________________________________

_______________________________________________

_______________________________________________

_______________________________________________

## 5) Anticipated failure modes & mitigations

**List** the top failure modes and link them to controls and scoring rationale.

For each failure mode, **complete all fields**:

1. Failure mode (mechanism): ____________________________
2. Potential real-world impact: (clinical harm, confusion, delay, privacy/identity misuse, equity gap)
3. Mitigation(s) in place: (guardrails, HITL review, disclosure, constrained generation, channel controls)
4. Residual likelihood term: Rare / Unlikely / Possible / Likely (after mitigations)
5. Residual severity term: Negligible / Minor / Major / Catastrophic (after mitigations)
6. Evidence/rationale: (artifact, pilot observation, precedent, expert judgement)

_______________________________________________

_______________________________________________

_______________________________________________

_______________________________________________

_______________________________________________

_______________________________________________

## 6) Ethical Alignment Score (EAS; 0/1/2 per principle)

**Score** each principle and cite supporting artifacts. **Note** uncertainty explicitly.

**Autonomy** (0/1/2): ___

Evidence: disclosure clarity; consent workflow; user control/opt-out; comprehension support.

_______________________________________________

**Beneficence** (0/1/2): ___

Evidence: expected benefit; alignment with guidelines; appropriateness for audience.

_______________________________________________

**Nonmaleficence** (0/1/2): ___

Evidence: mitigation strength; safety review; misuse resistance; escalation pathways.

_______________________________________________

**Justice** (0/1/2): ___

Evidence: language access; accessibility (captions); health-literacy provisions; equity plan.

_______________________________________________

**Total EAS** (0-8): ___

Key uncertainties/assumptions:

## 7) Residual risk tier

**Compute** residual risk as Likelihood × Severity **after mitigations** and **justify** the classification.

**Likelihood**: Rare / Unlikely / Possible / Likely

**Severity**: Negligible / Minor / Major / Catastrophic

**Residual risk tier**: ____________________________

**Rationale** (1-3 sentences):

_______________________________________________

_______________________________________________

_______________________________________________

_______________________________________________

## 8) Governance ask

**Request** a disposition and confirm minimum controls.

- Proposed disposition: Encourage / Permit with oversight / Restrict or redesign / Prohibit
- Minimum controls confirmed: disclosure; documentation; HITL (if applicable); provenance; monitoring; re-review triggers
- Time-limited approval requested? Yes/No. If yes, re-review date:

_______________________________________________

_______________________________________________

_______________________________________________

_______________________________________________

_______________________________________________

## 9) Monitoring plan & thresholds

**Specify** the monitoring plan as an operational commitment (who measures what, where, and when).

- Indicators (minimum set): comprehension; content-attributable follow-up burden; incidents/complaints; equity gaps; provenance/trust
- Data sources and owners: (portal analytics, messaging/call logs, incident/complaints system, feedback channel)
- Targets/baselines and review cadence: (weekly/monthly; minimum volume before interpreting gaps)
- Trigger thresholds: (signal excursion, major incident, equity flag, provenance/identity event)
- Re-review workflow: notified roles; response time; documentation required
- Decommission/recall criteria and override/escalation pathway:

_______________________________________________

_______________________________________________

_______________________________________________

_______________________________________________

_______________________________________________

_______________________________________________

_______________________________________________

## 10) Sign-offs

**Record** accountable reviewers and dates.

Clinician/Health educator: __________________ Date: ________

Bioethicist: _______________________________ Date: ________

Safety/IT lead: _____________________________ Date: ________

## Change log

**(versioned assumptions; rationale for deviations from precedent):**

For each version, **record at minimum**:

- Version & date: ________
- Change description: ________
- Rationale: ________
- Expected impact on risk/EAS: ________
- Evidence required before redeployment: ________
- Approver: ________
